# Adenomyoepithelioma of the breast: a challenging diagnosis on biopsy—case report and literature review

**DOI:** 10.1093/jscr/rjaf372

**Published:** 2025-06-12

**Authors:** Imane Tazi, Bouchra Kouhkouh, Soumaya Ech-charif, Ismail Boujida, Mouna Khmou, Youssef Mahdi, Basma El Khannoussi

**Affiliations:** Ibn Sina University Hospital Center Pathology, Rabat-Sale-Zemmour-Zaer, Rabat, Morocco; Faculty of Medicine and Pharmacy of Rabat Pathology, Mohammed V University of Rabat, Rabat-Sale-Zemmour-Zaer, Rabat, Morocco; Ibn Sina University Hospital Center Pathology, Rabat-Sale-Zemmour-Zaer, Rabat, Morocco; Faculty of Medicine and Pharmacy of Rabat Pathology, Mohammed V University of Rabat, Rabat-Sale-Zemmour-Zaer, Rabat, Morocco; Ibn Sina University Hospital Center Pathology, Rabat-Sale-Zemmour-Zaer, Rabat, Morocco; Faculty of Medicine and Pharmacy of Rabat Pathology, Mohammed V University of Rabat, Rabat-Sale-Zemmour-Zaer, Rabat, Morocco; Ibn Sina University Hospital Center Pathology, Rabat-Sale-Zemmour-Zaer, Rabat, Morocco; Faculty of Medicine and Pharmacy of Rabat Pathology, Mohammed V University of Rabat, Rabat-Sale-Zemmour-Zaer, Rabat, Morocco; Ibn Sina University Hospital Center Pathology, Rabat-Sale-Zemmour-Zaer, Rabat, Morocco; Faculty of Medicine and Pharmacy of Rabat Pathology, Mohammed V University of Rabat, Rabat-Sale-Zemmour-Zaer, Rabat, Morocco; Ibn Sina University Hospital Center Pathology, Rabat-Sale-Zemmour-Zaer, Rabat, Morocco; Faculty of Medicine and Pharmacy of Rabat Pathology, Mohammed V University of Rabat, Rabat-Sale-Zemmour-Zaer, Rabat, Morocco; Ibn Sina University Hospital Center Pathology, Rabat-Sale-Zemmour-Zaer, Rabat, Morocco; Faculty of Medicine and Pharmacy of Rabat Pathology, Mohammed V University of Rabat, Rabat-Sale-Zemmour-Zaer, Rabat, Morocco

**Keywords:** adenomyoepithelioma, breast tumor, core needle, case report

## Abstract

Adenomyoepithelioma of the breast (AME) is a rare tumor characterized by a dual proliferation of epithelial and myoepithelial cells. Although typically benign, AME carries a significant risk of recurrence and, in rare cases, malignant transformation. Its rarity, variable imaging features, and histological complexity make diagnosis challenging. Because it can closely mimic invasive breast carcinoma and overlap with other myoepithelial lesions, accurate histopathological assessment is essential. Here, we present the case of a 58-year-old woman with AME diagnosed on mastectomy following an initial misdiagnosis as carcinoma based on biopsy results. We review the literature and highlight the histopathological pitfalls associated with this rare entity.

## Introduction

Adenomyoepithelioma (AME) is a rare primary mammary neoplasm that is histologically characterized by biphasic proliferation of both epithelial and myoepithelial cells [1]. Alongside pleomorphic adenoma and malignant adenomyoepithelioma, they all belong to the epithelial-myoepithelial tumors [2]. Although these lesions are benign and have a good prognosis, some cases described in the literature have shown malignant transformation [3–5]. Diagnosis by simple biopsy remains difficult because of the diagnostic difficulties this entity can mimic. A mastectomy specimen is still required to rule out malignant transformation [6].

We report a case of adenomyoepithelioma confirmed through mastectomy specimen, following an initial misdiagnosis as carcinoma on biopsy. This article aims to emphasize the primary differential diagnosis, notably breast carcinoma, and to explore crucial factors that help prevent under diagnosis of this condition.

## Case presentation

A 58-year-old woman presented with a palpable right breast mass. Physical examination revealed a hard mass fixed to the skin in the upper outer quadrant. Ultrasonography showed a well-defined nodule categorized as BI-RADS 4B. MRI found a suspicious lesion with areola-nipple retraction, but no axillary lymphadenopathy.

An ultrasound-guided biopsy was performed outside our institution and misdiagnosed as a grade II invasive ductal carcinoma of no specific type (NST) according to the SBR (Scarff-Bloom-Richardson) grading system.

A mastectomy with axillary node dissection was performed. Gross examination revealed a well limited white tumor, measuring 30 × 25 mm, with no lymphadenopathy. Thin sections were prepared, and the tumor was entirely included for microscopic examination.

**Figure 1:**
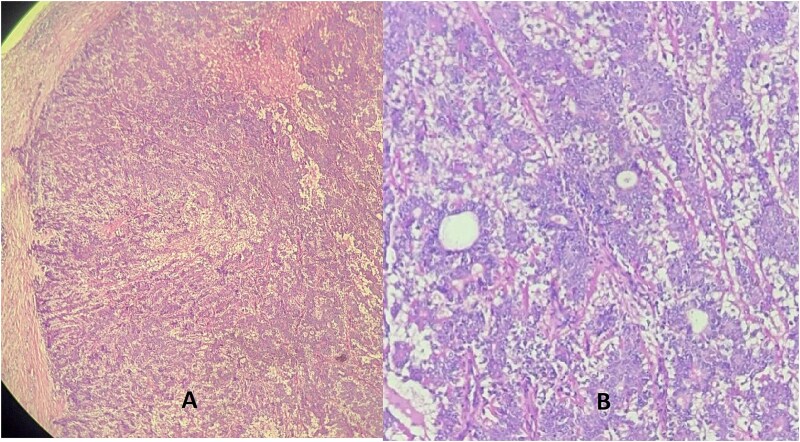
Hematoxylin eosin stain (A = ×10) (B = ×40) showing a well-defined tumor made of a dual component forming glands: One layer next to the glandular lumen = epithelial layer made of rounded cells with eosinophilic cytoplasm, resting on the myoepithelial layer whose cells are spindle-shaped with an elongated nucleus.

Microscopic examination revealed a well-circumscribed, non-encapsulated proliferation composed of biphasic epithelial structures forming glands. The luminal cells were eosinophilic with rounded nuclei, resting on a layer of spindle-shaped myoepithelial cells. No cytonuclear atypia or mitotic figures were observed (Fig. 1).

**Figure 2:**
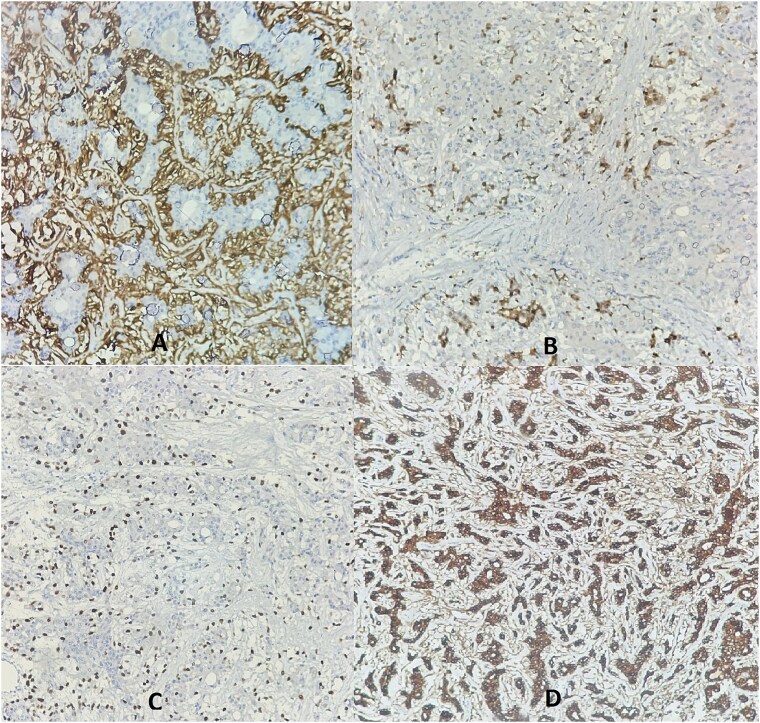
Immunohistochemistry revealed membranous staining for SMA (A), cytoplasmic staining for PS100 (B), and nuclear staining for P63 (C) in the myoepithelial layer cells, while the epithelial layer cells showed cytoplasmic staining for cytokeratin AE1/AE3 (D).

Additional immunohistochemistry revealed positive staining of myoepithelial cells with p63, smooth muscle actin (SMA), and S100 protein. The epithelial cells were positive for cytokeratin AE1/AE3 (Fig. 2).

**Figure 3:**
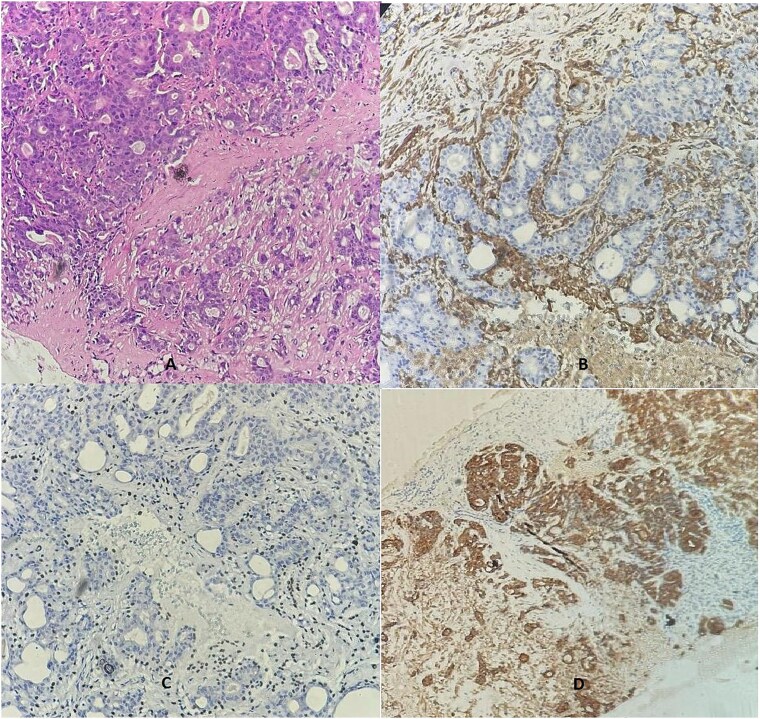
A breast biopsy core demonstrated dual cell proliferation (Ax10), confirming the presence of a basal layer with cytoplasmic staining for SMA (B) and nuclear P63 staining (C), alongside a luminal layer that stained positive for cytokeratin AE1/AE3 (D).

Due to discordance with the initial biopsy, it was re-examined internally, confirming the biphasic proliferation with similar immunostaining (Fig. 3). The final diagnosis was adenomyoepithelioma, and 4-month follow-up showed no recurrence.

## Discussion

Breast AME is a unique tumor that was first described by hamperl in 1970. While it most commonly arises in the skin, it can also occur in the mammary and salivary glands [7].

Due to its rarity and unusual presentation, it can mimic breast cancer on both the clinical and radiological levels [8].

The most common clinical presentation is a palpable mass without axillary adenopathy in women between the ages of 33 and 82 years [9, 10].

Sonography shows solid, hypoechoic, ovoid nodules with irregular or microlobulated borders. Mammography reveals oval or round isodense masses with clear borders [11]. MRI detects masses with round to uneven shapes, smooth or irregular margins, heterogeneous enhancement, and delayed washout [1]. Histological diagnosis remains the gold standard due to nonspecific imaging.

On gross examination, AME appears as a well-defined mass measuring between 0.3 and 8 cm, with a firm consistency, and a grayish-white color. It may sometimes exhibit hemorrhagic suffusion or cystic changes [12].

The histopathologic diagnosis of breast adenomyoepithelioma using core needle biopsy is difficult due to the tumor's inherent heterogeneity and the common issue of under-sampling associated with this biopsy method [1, 12].

Classically, adenomyoepithelioma presents as a well-defined glandular proliferation, often with a multilobulated contour and small peripheral satellite nodules, as described by the World Health Organization [2]. Tumor cells form a double layer: cuboidal luminal epithelial cells with eosinophilic cytoplasm, supported by polygonal or spindle-shaped myoepithelial cells, sometimes with clarified or plasmacytoid features [6].

Tavassoli described a variety of architectural patterns, including lobulated, papillary and tubular, usually in combination [13].

There is currently no consensus to distinguish between malignant or benign AME. The diagnosis of malignant adenomyoepithelioma is typically based on the presence of cytonuclear atypia and increased mitotic activity [5].

On IHC, the myoepiyhelial cells are positive for myoepithelial markers, such as smooth muscle actin (SMA), smooth muscle myosin heavy chain (SMMH), p63, S-100, CK5, CK14, H caldesmon, CD10, and naspin. The epithelial layer is positive for low-molecular-weight CK, such as CK7 and CK19, Epithelial membrane antigen (EMA) [10].

The diagnosis of adenomyepithelioma can easily be confused with invasive carcinoma, especially on core needle biopsy, as the luminal cells may show cytological atypia and may develop clusters that can mimic carcinoma [2]. It remains the most important differential diagnosis to rule out since it has significant clinical implications. Patients may be subjected to overtreatment, including total mastectomy, axillary lymph node dissection, and even adjuvant therapy, which may not be necessary for a benign tumor.

AME should also be differentiated from other benign breast lesions, especially nodular adenosis or intraductal papillomas. The myoepithelial cells present in AME are more abundant, thicker, and larger [2].

Total excision with adequate margins is recommended to prevent local recurrence. If recurrence occurs, more extensive excision may be needed. For benign adenomyoepitheliomas, mastectomy, breast-conserving surgery with radiotherapy, and axillary dissection are usually unnecessary. However, these treatments may be required if the adenomyoepithelioma progresses to cancer [12].

## Conclusion

To conclude, AME of the breast is a very rare condition that is often challenging to diagnose based on biopsy findings. The key takeaway is the potential for diagnostic confusion with invasive carcinoma, given that imaging alone is not definitive. A definitive diagnosis requires histological and immunohistochemical assessment following surgical removal of the tumor.

## Data Availability

Not applicable.
